# NADPH containing superoxide-producing thermostable complex from raspberry, apricot, grape, and grape seeds: isolation, purification, and properties

**DOI:** 10.1186/s13007-022-00978-9

**Published:** 2023-01-03

**Authors:** Sona M. Feschyan, Ruzan M. Simonyan, Gegham M. Simonyan, Maxim A. Simonyan, Ashkhen L. Manukyan

**Affiliations:** 1 Department of Biology, Yerevan State Medical University After M. Heratsi, Koryun 2, 0025 Yerevan, Armenia; 2 Laboratory of “Metabolism of Reactive Oxygen”, H. Buniatyan Institute of Biochemistry NAS RA, P.Sevag Str., 5/1, 014 Yerevan, Armenia; 3 Department of Medical Chemistry, Yerevan State Medical University After M. Heratsi, Koryun 2, 0025 Yerevan, Armenia

**Keywords:** Fe(III), Fruits, Hybrid associate, NADPH-containing protein, NADPH oxidase, Superoxide-producing complex

## Abstract

**Background:**

NADPH oxidase (Nox) plays a crucial role in reactive oxygen spices (ROS) production and mediates different diseases’ development. Under aerobic conditions, the NADPH-containing protein component of the (NCP)-Fe (III) complex produces O_2_^−^ continuously and intensively. However, after the removal of Fe (III), the isolated NCP shows only antioxidant properties at the expense of NADPH composite. Based on the fact that the mentioned fruit juices are widely used in everyday life and also in biomedicine, it was aimed to use a universal method: 1. to obtain superoxide generating complex from available and relatively cheap raw materials without using any toxic substances; 2. to isolate, purify and study the components of prooxidant nature: the isoforms of O_2_^−^—producing NCP-Fe (III) complexes obtained from Armenian fruits (raspberries, apricot, grapes), as well, grape seeds.

**Results:**

Using a licensed method, for the first time isoforms of the superoxide (O_2_^−^) producing a thermostable complex of the NCP component with Fe (III), (NCP-Fe (III)), were isolated and purified from Armenian fruits—raspberries, apricots, grapes, and grapes seeds. The process of isolation and purification of isoforms of these complexes included the following stages of processing: 1. alkaline hydrolysis at pH9,5; 2. their sedimentation at pH4.8; 3. Dissolving of the sediments in water at pH9.5, followed by ion-exchange chromatography on cellulose DE-52, and gel filtration on Sephadex G-100. Further, the heat treatment of the mentioned complexes was carried out. In a lyophilized state, under the anaerobic conditions, the isoforms of the given complexes, hybrid associates (hNCP-Nox), and NCP were stored practically without losing their activity in a mass of 1–1.5 g.

**Conclusions:**

Isoforms of O_2_^−^ -producing complexes are new liquid-phase, thermo-stable prooxidant components found in raspberries, apricots, grapes, and grapes seeds.

## Background

The isoforms of the cellular NADPH-dependent oxidases, collectively known as the Nox protein family, participate in different physiological and pathological processes in both the animal and plant kingdoms. Nox is a multimeric complex of the Nox family enzymes [1], first identified in phagocytic [2] and dendritic cells [3, 4]. It plays a crucial role in antimicrobial host defense. Congenital Nox deficiency leads to the development of chronic granulomatous disease, a primary immunodeficiency, characterized by life-threatening bacterial and fungal infections [5]. The role of NADPH oxidase in the pathogenesis of autoimmunity and the regulation of adaptive immune responses was determined [6]. Therefore, the function of Nox is important to direct the role of this enzyme towards tissue repair or increase resistance of the organism to oxidative stress [7].

NADPH-containing O_2_^−^ producing protein components and associates of NADPH—containing lipoprotein, as well as Nox (NLP-Nox) first isolated from the blood serum of mammals, erythrocyte ectosome and cellular membranes of medicinal plants, stevia [7–12]. The mechanism of O_2_^−^ production by these components is conditioned with electron transfer by associated ions of Fe (III) of the heme group of Nox isoforms from NCP to molecular oxygen, reducing it to O_2_^−^. However, NCP in fact is a new component of biomembranes. On the other hand, the membrane-associated formations (vacuoles) of fruit tissues (apricot, raspberry, grapes, grape seeds), significantly differ from those in cell membranes and intracellular formations of animals and plants by their density, strength, and functioning [13]. Indicated above fruits and grape seeds [14–16] have a certain antioxidant activity, due to which they have a protective effect observed in various types of pathological conditions and diseases [17–19]. As active intermediates, these fruits, as well as grape seeds contain ions of various metals, particularly iron and copper [20–22]. Evidently, the antioxidant status of fruits and seeds should be balanced with the prooxidant status.

Based on the fact that the mentioned fruit juices are widely used in everyday life and also in biomedicine, it was aimed to use a universal method: 1. to obtain superoxide generating complex from available and relatively cheap raw materials without using any toxic substances; 2. to isolate, purify and study the components of prooxidant nature: the isoforms of O_2_^−^—producing NCP-Fe (III) complexes from Armenian fruits (raspberries, apricot, grapes), as well, the grape seeds; 3. to separate from isoforms of these complexes of NCP and to determine the mechanisms of stimulation of O_2_^−^ production by NCP in hybrid associate in Nox of immune cells (leukocytes membranes (LM) and erythrocytes membranes (EM)) –hNCP-Nox.

## Results

### Purification of the isoforms of the NCP-Fe (III) complex from raspberries, apricots, grapes, and the grapes seeds

As a result of ion-exchange chromatography on a cellulose DE-52 column at a balanced pH of 9.5, fractions of isoforms of the NCP-Fe (III) complex obtained from raspberries, apricots grapes, and the grape seeds were not adsorbed; but eluted without delay. After concentration by vacuum lyophilization of the isoform of the NCP-Fe (III) complex and gel filtration on a column with Sephadex G-100, still, at pH 9.5, the first effluent fractions of the NCP-Fe (III) isoforms were collected and reunited with a symmetric elution diagram. To remove possible traces of protein components, aqueous solutions of NCP-Fe (III) isoforms were subjected to high-temperature treatment (heating in boiled water for 10–12 min), with further centrifugation. During electrophoresis on 10% PAAG, the above complexes did not pass through the tubes with PAAG and aggregated at the inlet of this gel. However, no stripes of protein amid-black staining on PAAG for the detection of the acidic and basic character of water-soluble proteins had been detected. Being based on: 1) the symmetry of the elution diagram of NCP-Fe (III) isoforms through G-100; 2) the absence of proteins staining band on PAAG; and 3) the constancy of the optical spectral purity index for isoforms of the NCP-Fe (III) complex got from raspberries (A280/A490), apricot (A280/A460), grape (A280/A420) and the grape seeds were: 8.1 ± 0.01; 7.8 ± 0.02 and 8.0 ± 0.01 and 8.4 ± 0,03, respectively (n = 6), we can indirectly infer about the purity of these complexes.

**Fig. 1:**
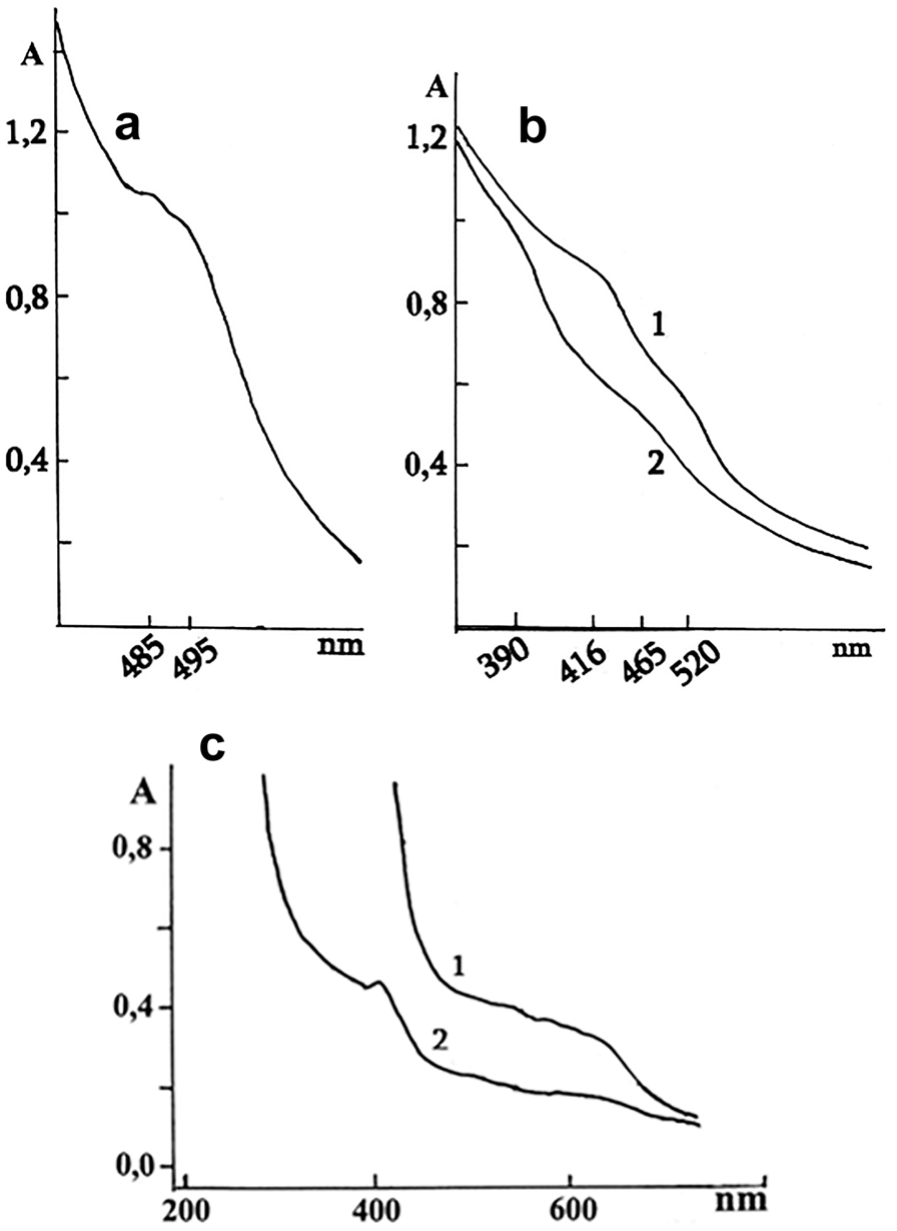
The optical absorption spectra of the isoforms of the NCP-Fe (III) complex were obtained from raspberries, apricots, grapes, and the grape seeds. Optical absorption spectra of weakly opalescent aqueous solutions of isoforms of the O_2_^−^-producing complexes NCP-Fe (III) obtained from raspberries **a**, grapes (**b**-1), apricots (**b**-2), and grape seeds (**c** 1,2) at pH9.5. In the UV region, there is an absorbance characteristic for proteins at 260–280 nm

After purification performed by the above-mentioned method, the optical absorption spectra of the isoforms of the NCP-Fe (III) complex obtained from raspberries, apricots, grapes, and the grapes seeds in the visible region differed significantly in the optical absorption maxima (Fig. 1).

### Determination of some quantitative characteristics of NCP-Fe (III) complexes from fruits and the grape seeds

**Fig. 2:**
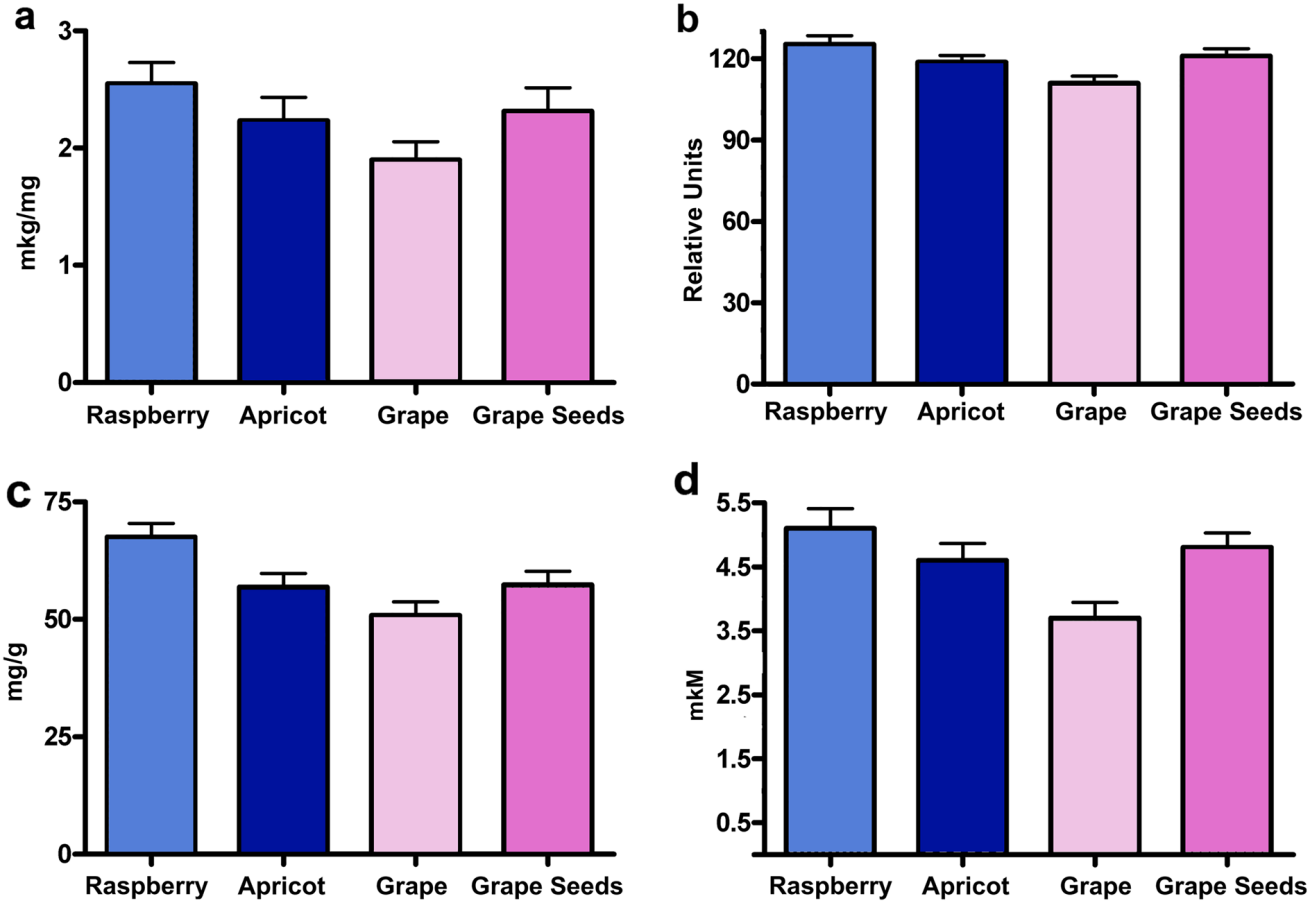
The content of Fe (III), spectrofluorimetric indices («F»), specific amounts of these complexes, and stationary concentration produced O_2_^−^. Content of Fe (III) (mkM/mg) in complexes **a**, «F» NADPH in relative units in complexes **b**, specific amount (mg/g) of complexes **c**, and the stationary concentration (mkM) of produced O_2_^−^ by 1 mg complexes, during 5 min at 25 °C **d** from fruits and seeds (n = 6)

In fact, in these fruits, the content of NADPH in the NCP-Fe (III) complexes was much higher than that of the NADPH-containing superoxide-producing lipoprotein serum of the placental blood of women (suprol) [6]. Indeed, there is a direct correlation between the NADPH content (Fig. 2) and the intensity of O_2_^−^ -production by the isoforms of the above-mentioned complexes.

### Oxidation of adrenaline to adrenochrome by the NCP-Fe (III) produced O_2_^−^

**Fig. 3:**
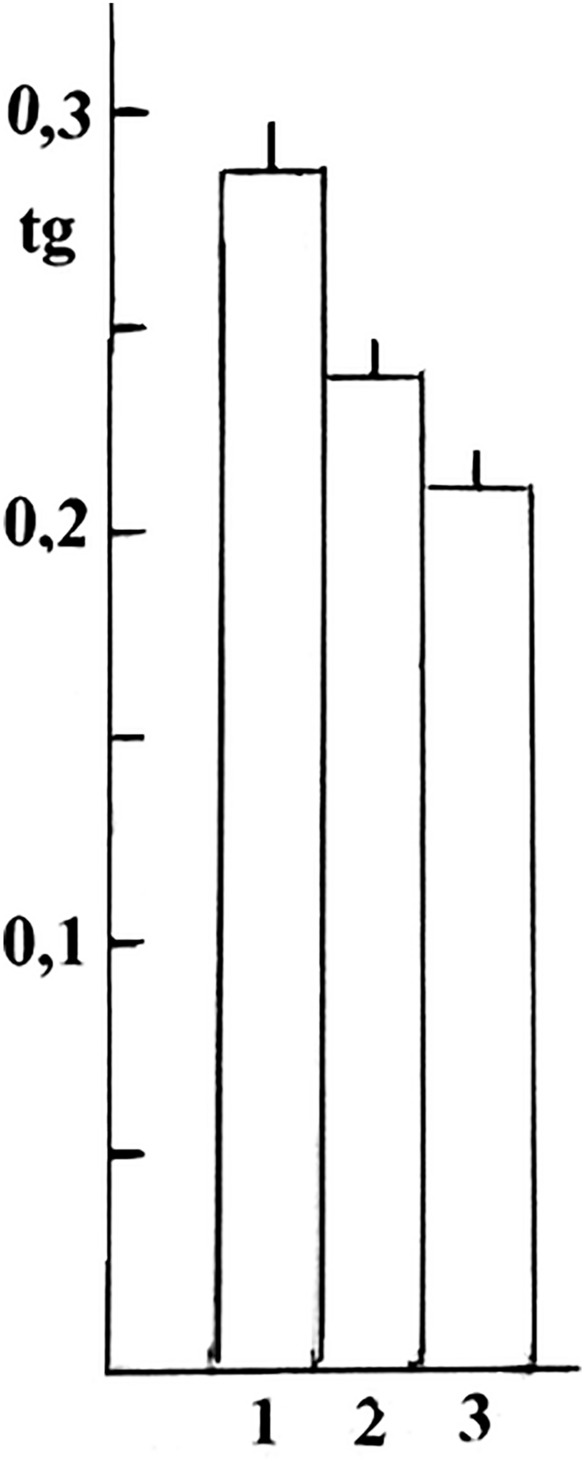
tg slope of the angle of kinetic curves of adrenochrome formation. The rate of adrenaline oxidation (4.10^–4^ M) into adrenochrome (tg slope of the angle of kinetic curves of adrenochrome formation at 500 nm) under the influence of O_2_^−^, produced in 0,2 mg/ml by isoforms of the NCP-Fe (III) complex got from raspberries (**1**), apricot (**2**) and grapes (**3**), at 25 °C, for 5 min

Direct correlation between the NADPH content and the intensity of O_2_^−^ production by the of NCP-Fe (III) complexes isoforms corresponds to an increase in density of the maximum optical absorption of adrenochrome as a result of adrenaline oxidation by O_2_^−^ produced by these complexes (Fig. 3).

As shown in Fig. 3, the direct correlation between the rate of adrenochrome formation with the NADPH content in the above-mentioned NCP-Fe (III) complexes obtained from raspberries, apricots, and grapes was detected.

### The steady-state concentration of O_2_^−^

**Fig. 4:**
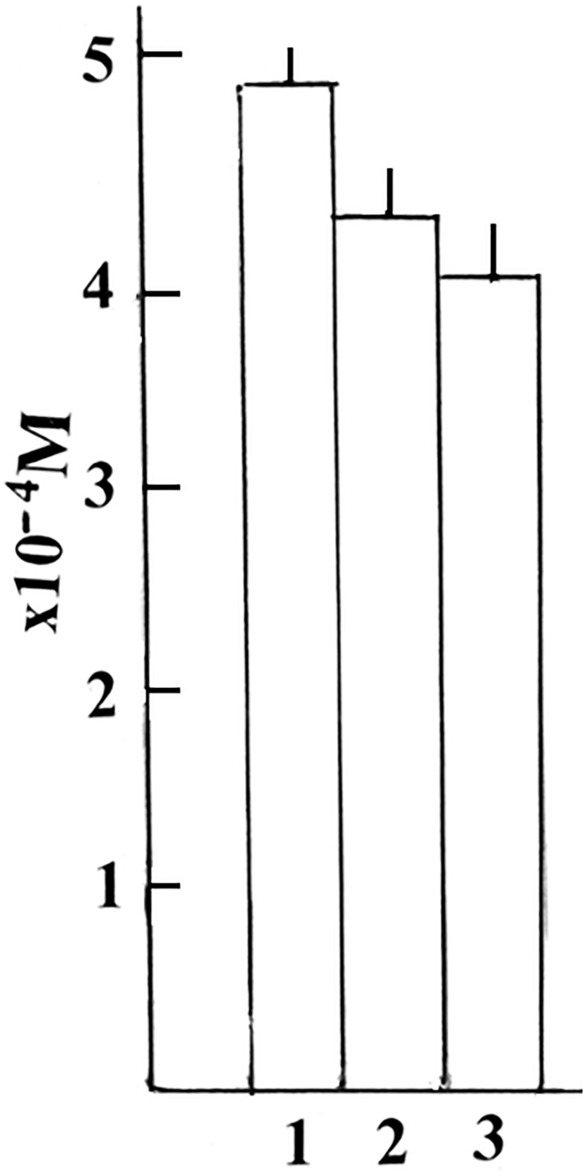
The steady-state concentration of O_2_^−^. The calculated stationary concentration (M) of O_2_.^−^ produced by isoforms NCP-Fe (III) got from 0, 2 mg/ml of raspberries (**1**), apricots (**2**), and grapes (**3**), (n = 6)

In fact, the steady-state concentration of O_2_^−^ produced was higher in the complex from raspberries, comparably to apricots and grapes (Fig. 4).

### Oxidation of adrenaline by superoxide radicals and reduction of potassium permanganate

**Fig. 5:**
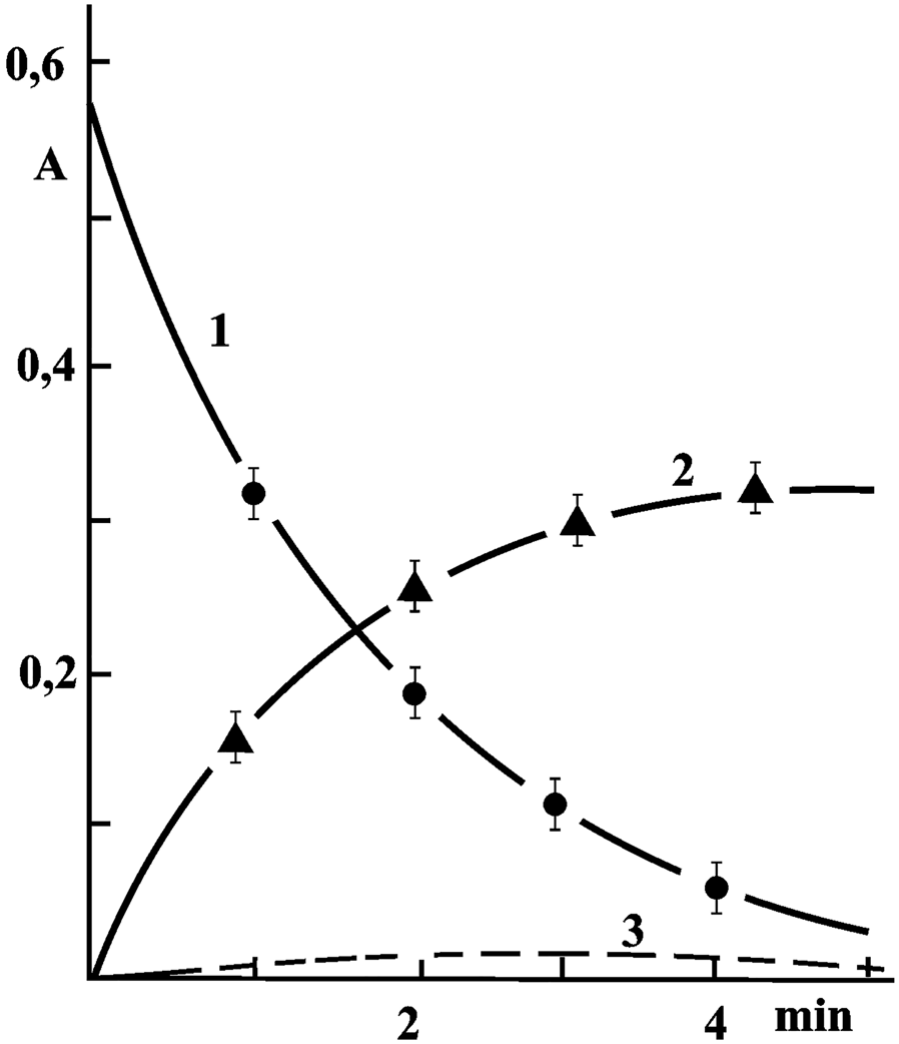
The kinetic curves of the reduction of potassium permanganate. The kinetic curves of the reduction of potassium permanganate (A530 = 0.8) by 0.2 mg/ml NADPH containing protein component—NCP (**1**); of oxidation of adrenaline (4.10.^−4^ M) into adrenochrome by superoxide radicals produced, in particular, by the NCP-Fe (III) isoform from raspberries (**2**). Curve (**3**) is identical to the curve (**2**) in the presence of NCP at 25 °C (n = 6)

**Owing** to the NADPH electron, NCP had only a reducing (antioxidant) effect, preventing the oxidation of adrenaline by superoxide radicals and reducing potassium permanganate, as it is shown in Fig. 5.

### Isolation and purification of Nox1+Nox2 from EM and LM

**Fig. 6:**
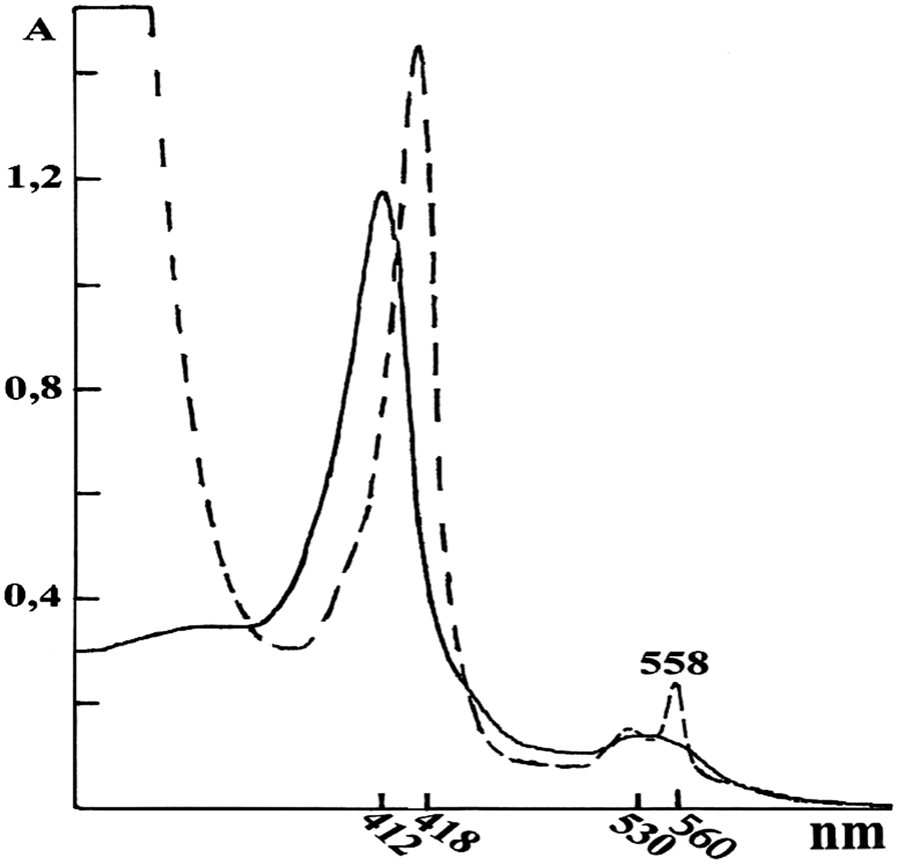
Absorbance optical spectra of the total fraction of Nox1+Nox2 isoforms got from EM and LM in oxidized and reduced states with absorption typical to Nox at 558 nm. Optical absorbance spectra of total fraction of Nox1+Nox2 obtained from EM or LM (___) of donor blood at pH7.4. After a reduction of NOX with sodium dithionite, acute absorbance, typical for Nox at 558 nm (––) appeared

Homogenized by electrophoresis total fractions of the isoforms of Nox1+Nox2 from EM and LM were isolated and purified by licensed methods using the recently discovered phenomenon of unstable complex formation of ferrihemoglobin with Nox isoforms and their release from biomembranes into a soluble phase (Fig. 6).

The obtained NCP-Fe (III) complexes produce O_2_^−^ by using Fe (III) as an electron carrier from NADPH in its composition to the molecular oxygen, reducing it up to O_2_^−^, which oxidizes adrenaline up to adrenochrome or reduces NTB up to formazan. These processes are inhibited by 2.10^−8^ M Cu, Zn-SOD.

### Formation of adrenochrome from grapes seeds under the influence of NCP

Forming the hybrid associates of isoforms of NCP and Nox from EM and LM (hNCP-Nox), the NCP, using Fe (III) of heme group of the Nox isoforms from EM and LM (as an electronic bridge) transfer the electron from NADPH to O_2_, reducing it up to O_2_^−^ in both homogeneous and heterogeneous phases. The increase of the superoxide-producing activity, in particular, by NCP from grape seeds, the superoxide-producing activity of EM and LM in homogeneous and heterogeneous phases is observed (Table 1).

**Table 1:** The increased percentage of formed adrenochrome under the influence of NCP from the grapes seeds in the homogeneous and heterogeneous phases, compared to 100% control indices (indices in the absence of NCP)

Parameters	O_2_^−^ producing components in the presence of 0.2 mg/ml NCP (control)	O_2_^−^ producing components in the presence of 0.2 mg/ml NCP from grape seeds (experimental group)	Percentage increase of maximal optical absorption density of adrenochrome at 500 nm
Nox1 + Nox2 (0.2 mg/ml) of EM	0.12 ± 0.012	0,19 ± 0.011***	58.4
Nox1+ Nox2 (0.2 mg/ml) of LM	0.14 ± 0.012	0,23 ± 0.027*	64.2
EM (0.5 mg/ml)	0.25 ± 0.017	0,43 ± 0.02***	72
LM (0.5 mg/ml)	0.31 ± 0.018	0,56 ± 0.02***	80.6

The statistical difference is provided between the control and experimental groups (n = 6),

(*P < 0.05, **P < 0.01, ***P < 0.001)

Our data have shown a relatively increased percentage of Nox1+Nox2 due to NCP from the grape seeds, the EM (p < 0.01), and the LM (p < 0.001) in the homogeneous phase of NTB reduction by superoxide radicals compared to control. In the heterogeneous phase, the NCP from the grape seeds and the increased percentage of Nox1+Nox2 in EM (p < 0.001) and LM (p < 0.001) was determined compared to the control.

In the isoforms of hNCP-Nox associates, the determining amount of the traces of Nox is presented. On the optical absorbance spectra of these isoforms of hNCP—Nox associates were observed weak characteristic absorbance maximums of the Nox (560 nm, 530 nm, and 412 nm), together with the characteristic maximal absorbances of these hybrid associates.

## Discussion

The O_2_^−^-producing complex of NADPH-containing protein component with Fe (III) was isolated and purified from Armenian fruits: raspberries, apricots, grapes, and grape seeds for the first time. The high thermostability of these complexes is quite possible, conditioned with the action at the high-temperature metabolic process (to 280 °C, at nanosecond) for some proteins (catalase, peroxidases, etc.).

The NADPH oxidase (Nox)-mediated release of ROS in oxidative burst represents it as an inflammatory mediator [4]. Over time, superoxide generation through Nox was thought to occur only in phagocytes; however, several enzymes responsible for ROS production have been recently identified in various tissues. These enzymes show similarities to phagocyte Nox and are collectively referred to as the Nox family. The Nox genes produce the transmembrane proteins responsible for transporting electrons across biological membranes, thereby the reduction of oxygen into superoxide [5]. Nox enzymes mediate different functions in various organisms through redox signaling. The importance of ROS in host immunity has been clearly determined in the genetic disorder, CGD, which reflects defects in Nox [23] and results in improper neutrophil killing during phagocytosis [24, 25].

Actually, by changing the concentration of isoforms of NCP-Fe (III) complexes and other conditions (temperature, time) of the reaction, it is possible to regulate the steady-state concentration of the produced O_2_^−^. This testifies about the controllability of the steady-state concentration of O_2_^−^ in the above biological systems, when determining the effect of the produced O_2_^−^ in empirically low and high concentrations on the process of proliferation and apoptosis of cells, as well as determining the antibacterial, anticancer and antiviral effects of O_2_^−^ produced by these thermo-stable systems [26–29].

However, after the association of NCP with the total fraction of Nox isoforms (Nox1+Nox2) obtained from the membranes of erythrocytes and leukocytes of donor blood, the formed hybrid associates (0.2 mg/ml each) oxidize adrenaline to adrenochrome almost at intensity that it took place under the action of isoforms NCP-Fe (III). It is possible that in case of immunodeficiency caused by a decrease in the O_2_^−^ -producing activity of leukocytes Nox, the NCP isoforms can increase the O_2_^−^—producing activity of Nox on the surface of leukocytes membranes [30].

For the first time, the mechanism of direct production of O_2_^−^ by the isoforms of NCP-Fe (III) from these fruits and the grape seeds’ isoforms when passing an electron from the NADPH group of NCP to Fe (III), followed by transfer to O_2_, thereby reducing it to O_2_^−^ had been revealed. Isoforms of the NCP-Fe (III) complex continuously produced O_2_^−^ under aerobic conditions. After the removal of Fe (III) ions from the NCP-Fe (III) complex, NCP isoforms exhibited only a reducing effect and stimulated the production of O_2_^−^ by isoforms of NADPH oxidases (Nox), in particular, of Nox from the membranes of immune cells (erythrocytes and leukocytes) by creating a hybrid associate (hNCP-Nox).

It is well known, that in systems for the production of O_2_^−^, in particular, during the depletion of hydrogen peroxide with alkali (KOH) [31] or ammonia [32], along with O_2_^−^ the highly toxic to biosystems hydroxyl radicals are formed; beyond this, hydrogen peroxide is also available, the toxicity of which is known rather well. The produced O_2_^−^ by isoforms of the NCP-Fe (III) complex or by the hybrid associate hNCP-Nox obtained from raspberries, apricots, grape, and the grape seeds were practically pure and did not contain other reactive oxygen species. NCP-Fe (III) and the hybrid associate hNCP-Nox continuously and intensively produced O_2_^−^ in the controlled steady-state concentrations. This fact seems to be more promising for determining the molecular mechanisms of action by the controlled steady-state concentrations of O_2_^−^ on various biosystems, considering their high thermo-stability (they were not inactivated when heated in boiled water 8–10 min), which gives birth to real prospects for determining the mechanisms of redox metabolic processes with the participation of O_2_^−^ at the high temperatures.

According to our data, a relatively increased activity of Nox1 + Nox2 in the processes of adrenaline oxidation in EM (58.4%, 66.7%) and LM (64.2%, 69%), as well as of formazan reduction in EM (72%, 74%) and LM (80.6%, 82.8%) was observed both in the homogeneous and heterogeneous phases due to the presence of the grapes seeds.

Some increase in the stationary concentration of O_2_^−^ in the heterogeneous phase is related to the fact that on the surface of EM or LM there is already located the O_2_^−^ producing Nox1 + Nox2 associated with NADPH containing lipoprotein which constantly produces O_2_^−^ under the aerobic conditions [6]. At the same time under the above-mentioned conditions, O_2_^−^- producing activity of leukocytes Nox is more (3–4 times) than that of the erythrocytes Nox, however, the content of erythrocytes is far superior to that of leukocytes. At the same time, erythrocytes like leukocytes are also components of the immune system [33].

Thus, there are some perspectives for using the NCP-Fe (III) complex from the grape seeds as an energetic, natural, and relatively stable O_2_^−^ production system, and so a natural bactericidal and antiviral agent [26]. NCP obtained from the mentioned sources can be used as a natural agent for decreasing O_2_^−^ producing activity of Nox isoforms of EM and LM in weakened immunity (immune deficiency) of mammals [30] experimentally and clinically in perspective*.*

As we have already mentioned above, these NCP-Fe (III) complexes in a lyophilized state, especially in the nitrogen presence, practically don’t lose their O_2_^−^-producing activity when kept at −10 to−15^0^C throughout the year. Loss of activity is not observed after the dissolution of NCP-Fe (III) or NCP in в 0.01 M PPB (pH7.4) with saline either. It serves as an opportunity to introduce NCP into the blood of animals. It should be noted that an analogous agent (suprol) from the females’ placenta blood did not cause any adverse unfavorable effects after injection into white rats even in much more amounts than in the norm, and moreover, the introduced suprol has an antitumor effect [34, 35].

In a lyophilized state, under the anaerobic conditions, the isoforms of the given complexes, hybrid associates, and NCP were stored practically without losing their O_2_^−^-producing activity in 1–1.5 years.

## Conclusion

It can be concluded that isoforms of new prooxidant complexes (O_2_^−^ -producing) (as a counterbalance to the antioxidant status) of NCP and Fe (III), isoforms of the antioxidant component of NCP, as well as the isoforms of a hybrid associate of NCP and Nox of immune cells, quantitative and qualitative changes in which they can be used as new markers for assessing the above-mentioned fruits grown in various environmental and ecological conditions. In addition, O_2_^−^—producing complexes NCP-Fe (III), isolated for the first time from grapes seeds as well as NCP, as activators of Nox of the immune system cells, are natural, with easily adjustable concentrations and new agents for the active oxygen metabolism regulation.

In conclusion, we can note that unlike Nox, which takes electrons from NADPH in the cytosol, NADPH is present in our fruit associates and complexes. The latter is a "biological automaton" transferring its own electrons to oxygen, and thus, it is a constantly operating mechanism.

It is planned to generate gas-phase monocomponent superoxide radicals of fruit and seeds associates which can be used in the clinic together with oxygen masks for the treatment of infections and pneumonia.

## Methods

During our research, for the first time, an NADPH-containing protein was isolated. The problem we set was to clarify the mechanism by which NADPH-containing proteins produce superoxide radicals. Therefore, NADPH has been identified as a possible source of electrons, which by some mechanism can regenerate the oxygen molecule by converting it into a superoxide radical. Therefore, the performed experiments were aimed at determining NADPH and elucidating the mechanism of superoxide radical formation.

### Isolation and purification of erythrocyte and leukocytes’ membranes from donor blood

After centrifugation of the II group donor blood (n = 6, 20 ml each) the precipitated leukocytes were isolated from erythrocytes by using 3% Dextran-70 (“Loba Finchemie”, Germany) dissolved in saline solution [36]. After washing the erythrocytes’ precipitates with 0.9% NaCl and their hemolysis in water, EM were sedimented by centrifugation at 5800 ×*g*, 10 min (centrifuge K-70, Janetzki, Germany), at pH 5,6. Then, EM was washed with 0.04 M potassium phosphate buffer, at pH7.4 (PPB) and precipitated under the above-mentioned conditions until colorless supernatant was obtained. Leukocytes from the donor blood were precipitated by centrifugation under analogical conditions. After washing the leukocytes’ precipitate was homogenized by adding water, and after freezing and thawing the LM were precipitated also at pH5.6 by centrifugation in similar conditions. Followed by washing with 0.04 M PPB, the LM precipitate was collected by centrifugation 13,000 ×*g*, 10 min, (centrifuge K-24). The obtained EM and LM sediments were mixed with water (1:10 v/v). The blood serum was additionally centrifugated by 14,000 ×*g*, 15 min.

### Isolation and purification of total fraction of Nox1 + Nox2 isoforms from EM and LM

In aqueous mixtures, EM and LM were adjusted to pH 9.5 by the addition of 0.1 M KOH, as well as 50mkM ferrihemoglobin (Hb) of the human erythrocytes and were incubated at 37 °C for 1.5 h. After centrifuging at 5800 ×*g* for 10 min the supernatants were exposed to ion-exchange chromatography in columns with DE-52 cellulose. After the eluation of the Hb fraction by 0.005 M PPB the total fractions of Nox1+Nox2 isoforms of EM and LM were eluted by 0.2 M PPB. After gel-filtration of the total fraction of Nox1+Nox2 isoforms in the column with Sephadex G-100, the traces of Hb included in the complex with Nox were removed by ethanol/chloroform fractionation [37].

### Isolation and purification of isoforms of the NCP-Fe (III) complex from raspberries, apricots, grapes, and grape seeds

The isoforms of O_2_^−^—producing NCP-Fe (III) complexes from the above-mentioned fruits and seeds were isolated and purified by licensed methods [38], whit the process of releasing the isoforms of NCP-Fe (III) complexes at pH9,5 by ferriHb (50 mkM) from indicated fluids, and precipitation at pH4.8 and solubilization in the water at pH9,5. The further process of purification of these complexes fractions included ion-exchanging chromatography on cellulose DE-52 and gel filtration on Sephadex G-100 at pH 9.5. Then, for the removal of the other proteins traces, the thermal treatment of water solutions of indicated above complexes was performed by heating in boiling water during 10–12 min.

**Electrophoresis** of the obtained complexes and hybrid associates was performed in 10% polyacrylamide gel (PAAG) for the proteins of acidic and basic character. The specific content (mg/g) of NCP-Fe (III), hNCP-Nox, as well as Nox from EM and LM was determined by weighing after their desalting and vacuum lyophilization.

### Separation of NCP by removing Fe (III) from isoforms of the NCP-Fe (III) complex

After incubation of EDTA (0.05 M) with NCP-Fe (III) complex (0.5 mg/ml) at 37 °C for 25–30 min, the mixture underwent ion-exchange chromatography on DE-52 cellulose, at pH9.5. Under these conditions, EDTA with Fe (III) was retained on the column, and NCP is eluted without any delay.

### Formation of hybrid associate between isoforms of NCP with Nox (hNCP-Nox) from EM and LM

Isoforms of the listed NCP were incubated separately with Nox isoforms from EM and LM [38] in equal specific contents (4–5 mg/ml) at 37 °C for 25–30 min, and the ion-exchange chromatography was performed on a column of DE-52 cellulose at pH9.5. From this column, the isoforms of the hNCP-Nox were eluted immediately.

### Determination of NADPH in NCP-Fe (III) complex

NADPH group in the NCP-Fe (III) complex was determined by spectrofluorimetric method, using Perkin Elmer spectrofluorimeter (USA). The emission peak of the NADPH group as a part of NCP was registered at 430 nm with a 370 nm excitation length.

### Determination of the Fe (III) in the composition of isoforms of O_2_^−^-producing complexes

The Fe (III) in the NCP-Nox complexes was determined by an atomic absorption spectrometer [39].

### Determination of the rate of adrenaline oxidation into adrenochrome (tg of the slope of the kinetic curves) by isoforms of the NCP-Fe (III) complex and the associates of hNCP-Nox

The rate of oxidation of adrenaline (4.10^−4^ M) into adrenochrome (at 500 nm) under the influence of O_2_^−^—producing isoforms of the NCP-Fe (III) complex from raspberries, apricots, grapes, and the grapes seeds, as well as the hybrid associate hNCP-Nox (0.4 mg/ml), was determined in the homogeneous phase (in solution) at 20 °C for 5–6 min.

### Determination of the percentage increase of stationary concentration of the produced O_2_^−^

The stationary concentration of O_2_^−^ produced by NCP-Fe (III) complexes, Nox isoforms from EM and LM in the presence of NCP was determined by the adrenaline [40] and nitrotetrazolium blue (NTB) [41] methods. In the latter case phenazine, a methosulfate initiator (10^−5^ M) was added. The produced O_2_^−^ oxidized adrenaline (2.10^−4^ M) to adrenochrome (at 500 nm) and reduced NTB (4.10^−4^ M) to formazan (at 560 nm). The percentages of increase of maximal optical absorbance density of adrenochrome and formazan were determined by producing O_2_^−^ in the absence (100%) and presence of NCP. The stimulation of the O_2_^−^producing process by Nox1 + Nox2 isoforms in the homogenous phase and EM and LM in the heterogenous phase in the presence and absence of NCP was determined after the incubation of the mentioned Nox-es (0.2 mg/ml each from EM and LM), as well as EM and LM (2 mg/ml each) with the obtained NCP (2 mg/ml each) at 37 °C for 5 min. To suppress these processes the 2.10^–8^ M Cu, Zn-SOD was used.

### Determination of the steady-state concentration of produced O_2_^−^

The steady-state concentration of the produced O_2_^−^ in moles (M) was determined by the adrenaline method, calculating the ratio of the density of the maximum optical absorbance (A) of the formed adrenochrome (at 500 nm) to the molar absorbance of the adrenochrome (E = 750 M^−1^ cm^−1^): A/E, taking into account that 1 M adrenaline is oxidized by one mole of O_2_^−^ [40].

### Statistical analysis

Analysis was performed using the BIOSTA system. All measurements were represented as mean ± SEM. The significance of the means’ difference was evaluated using paired Student Newman—Keuls test. Statistical significance, determined by one‐way ANOVA, was set at P < 0.05 (*P < 0.05, **P < 0.01, ***P < 0.001).

## Data Availability

The datasets generated during and/or analyzed during the current study are available from the corresponding author on reasonable request.

## Abbreviations

ROS: Reactive oxygen spices
NCP: NADPH-containing protein component
hNCP-Nox: Hybrid associates
NLP-Nox: NADPH containing lipoprotein
EM: Erythrocytes’ membranes
PPB: Potassium phosphate buffer
LM: Leukocytes’ membranes
Hb: Ferrihemoglobin
PAAG: Polyacrylamide gel
NTB: Nitrotetrazolium blue
